# Interfacial atomic structure analysis at sub-angstrom resolution using aberration-corrected STEM

**DOI:** 10.1186/1556-276X-9-578

**Published:** 2014-10-17

**Authors:** Chien-Nan Hsiao, Shou-Yi Kuo, Fang-I Lai, Wei-Chun Chen

**Affiliations:** 1 Instrument Technology Research Center, National Applied Research Laboratories, Hsinchu 30076, Taiwan; 2 Department of Electronic Engineering, Chang Gung University, Gueishan 33302, Taiwan; 3 Department of Photonics Engineering, Yuan Ze University, Taoyuan 32003, Taiwan

**Keywords:** Aberration correction, HRTEM, HRSTEM, HAADF, *Z* contrast, EELS, EDS

## Abstract

The atomic structure of a SiGe/Si epitaxial interface grown via molecular beam epitaxy on a single crystal silicon substrate was investigated using an aberration-corrected scanning transmittance electron microscope equipped with a high-angle annular dark-field detector and an energy-dispersive spectrometer. The accuracy required for compensation of the various residual aberration coefficients to achieve sub-angstrom resolution with the electron optics system was also evaluated. It was found that the interfacial layer was composed of a silicon single crystal, connected coherently to epitaxial SiGe nanolaminates. In addition, the distance between the dumbbell structures of the Si and Ge atoms was approximately 0.136 nm at the SiGe/Si interface in the [110] orientation. The corresponding fast Fourier transform exhibited a sub-angstrom scale point resolution of 0.78 Å. Furthermore, the relative positions of the atoms in the chemical composition line scan signals could be directly interpreted from the corresponding incoherent high-angle annular dark-field image.

## Background

Controlling epitaxial interfaces at the atomic scale is actively being researched in the broad fields of materials science and device engineering. To resolve and identify the atoms in materials with atomic-scale resolution has been a longstanding goal of analytical microscopy. The point resolution of high-resolution transmission electron microscopy (HRTEM), however, has been limited for over 70 years by the unavoidable lens aberration [1,2] and radiation damage that occurs at high acceleration voltages. Aberrations for an electronic optics system are introduced in the aberration function *χ*(*θ*, *φ*) which is the error in the optical path length between a perfect planar wave and the actual wavefront as a function of the diffraction angles *θ* (radial) and *φ* (azimuthal). The notation by Haider et al. is often used, and the aberration function is as follows:

$$\chi \left(\theta ,\varphi \right)=\sum_{\begin{array}{l} m<n+1\\ m+\text{nodd} \end{array}}\frac{1}{\left(m+n\right)}\left|C_{n,m}\right|\theta^{n+1}\text{cos}\left(m\varphi -\varphi_{C_{n,m}}\right)\text{.}$$

An aberration of order *n* and angular multiplicity *m* is characterized by an amplitude |*C*_*n*,*m*_| and a phase $\varphi_{C_{n,m}}$:

$$\begin{array}{ll} \chi \left(\theta ,\varphi \right)= & \frac{1}{2}A_{1}\theta^{2}\text{cos}\left(2\varphi -\varphi_{A_{1}}\right)+\frac{1}{2}C_{1}\theta^{2}+\frac{1}{3}A_{2}\theta^{3}\text{cos}\left(3\varphi -\varphi_{A_{2}}\right)\\ & +B_{2}\theta^{3}\text{cos}\left(\varphi -\varphi_{B_{2}}\right)+\frac{1}{4}C_{3}\theta^{4}+\frac{1}{4}A_{3}\theta^{4}\text{cos}\left(4\varphi -\varphi_{A_{3}}\right)\\ & +S_{3}\theta^{4}\text{cos}\left(2\varphi -\varphi_{S_{3}}\right)+\ldots , \end{array}$$

where *A*_1_, *C*_1_, *A*_2_, *B*_2_, *C*_3_, *A*_3_, and *S*_3_ are, respectively, the second-order astigmatism, defocus, third-order astigmatism, coma, spherical aberration (i.e., Cs), fourth-order astigmatism, and star aberration.

Recently developed aberration-corrected HRTEM [3,4] and high-resolution scanning transmission electron microscopes (HRSTEM) improve the resolving power without the need to increase the electron beam energy [5-8]. Although still far from their ultimate diffraction limit, these instruments have been demonstrated to resolve sub-angstrom image features. HRSTEM high-angle annular dark-field (HAADF) imaging can be considered incoherent, thus nearly completely eliminating the diffraction and phase contrast. The contrast is then, to a good approximation, monotonic with thickness, and is also sensitive to changes in composition; for a typical geometry and material, it is approximately proportional to *Z*^1.8^, where *Z* is the atomic number. These properties also make the STEM HAADF signal ideal for recent tomography applications [9-11]. However, although many literature reports describe the instrumental details of how the aberration correctors are designed and function, the practical aberration correction for the atomic structure determination and interface analysis of functional materials is also important. On the other hand, the development of a device that can measure the distribution of all of the elements present in the material structure in order to monitor the success and quality of the process is required. To date, electron energy loss spectroscopy (EELS) [12,13] and energy distribution spectroscopy (EDS) [14,15] have been shown to be effective for measuring not only images of atoms but also chemical composition. The higher resolution provides much improved sensitivity for the atomic arrangements at defects and interfaces. Combining EDS with HRSTEM offers two advantages: inelastic interactions are always effectively local, such as in an incoherent HAADF image, and the inner shell ionization potential is as localized as possible for a given ionization edge. Consequently, the resulting incoherent image can be directly interpreted [15]. In the present study, atomic resolution HRSTEM images of SiGe/Si interface were directly interpreted along with simultaneous chemical line scans based on detection of the incoherent signal using an aberration-corrected scanning transmission electron microscope.

## Methods

Electropolished and chemically etched specimen of an epitaxial SiGe/Si semiconductor grown using molecular beam epitaxy (MBE) on a single crystal silicon [001] substrate was used in this study. An aberration-corrected STEM equipped with an HAADF detector and an EDS was used to analyze the atomic structure of each specimen (Titan 200 kV). Au-Pd bimetallic particles deposited on a thin amorphous carbon film was used as a reference for correcting the aberration coefficients of the electronic optics system. Because the aberrations were visible when the beam was tilted out of the optical axis, a series of images was acquired at different beam tilt angles [3]. The aberration correction process was principally achieved by calculating the Zemlin tableau of a series of aberration coefficients followed by visualization of the phase shift image (phase plate). The required accuracy for compensation of the various residual aberration coefficients needed to achieve sub-angstrom resolution with the system was then determined. The probe forming aperture semiangle was 9.8 mrad, and the probe was focused to the size of 1 Å on the specimen with a beam current of 70 pA. The EDS was used to detect the chemical element distribution of the SiGe/Si interface. In this method, a coherent focused probe was scanned across the specimen, and the resultant x-ray emission spectrum was recorded at each probe position. These spectra were then used to construct an elemental line scan. The acquisition time for a single x-ray spectrum was 20 s.

## Results and discussion

### Aberration correction

The calculation for an electron beam profile consisted of the calculation for the propagation of an electron wave through a phase plate and the calculation for the intensity of the resulting wave. In addition, the probe profiles were determined assuming that the wave function in the object plane for a fixed electron energy can be calculated as the Fourier transform of the phase shifts in the aperture plane. The phase shift is caused by the various coherent axial aberrations *A*_1_ up to *C*_5_ (and higher order aberrations). Furthermore, a Gaussian distribution of the electron energies was assumed:

$$\frac{dP\left(E\right)}{dE}=\frac{1}{\sigma \sqrt{2\pi }}\text{exp}\left(\frac{-\left(E-E_{0}\right)}{2\sigma^{2}}\right),$$

where *σ* is the standard deviation of the energy distribution, *E*_0_ is the mean value of the primary energy, and *E* is the actual energy of one electron. The full-width half-maximum (FWHM) was also calculated as *ΔE*_*E*FWHM_ = (8 ln 2)^1/2^*σ*. The complete intensity distribution *J*_*t*_(*w*) is then given as an incoherent superposition of the distributions for the individual energies:

$$J_{t}\left(w\right)={\int_{-\infty }^{\infty }\left|\psi \left(w,E\right)\right|}^{2}\frac{dP}{dE}dE.$$

According to this calculation, the influence of the various axial aberrations on the shape and even more importantly on the electron intensity distribution within the probe could be evaluated [6].

**Figure 1:**
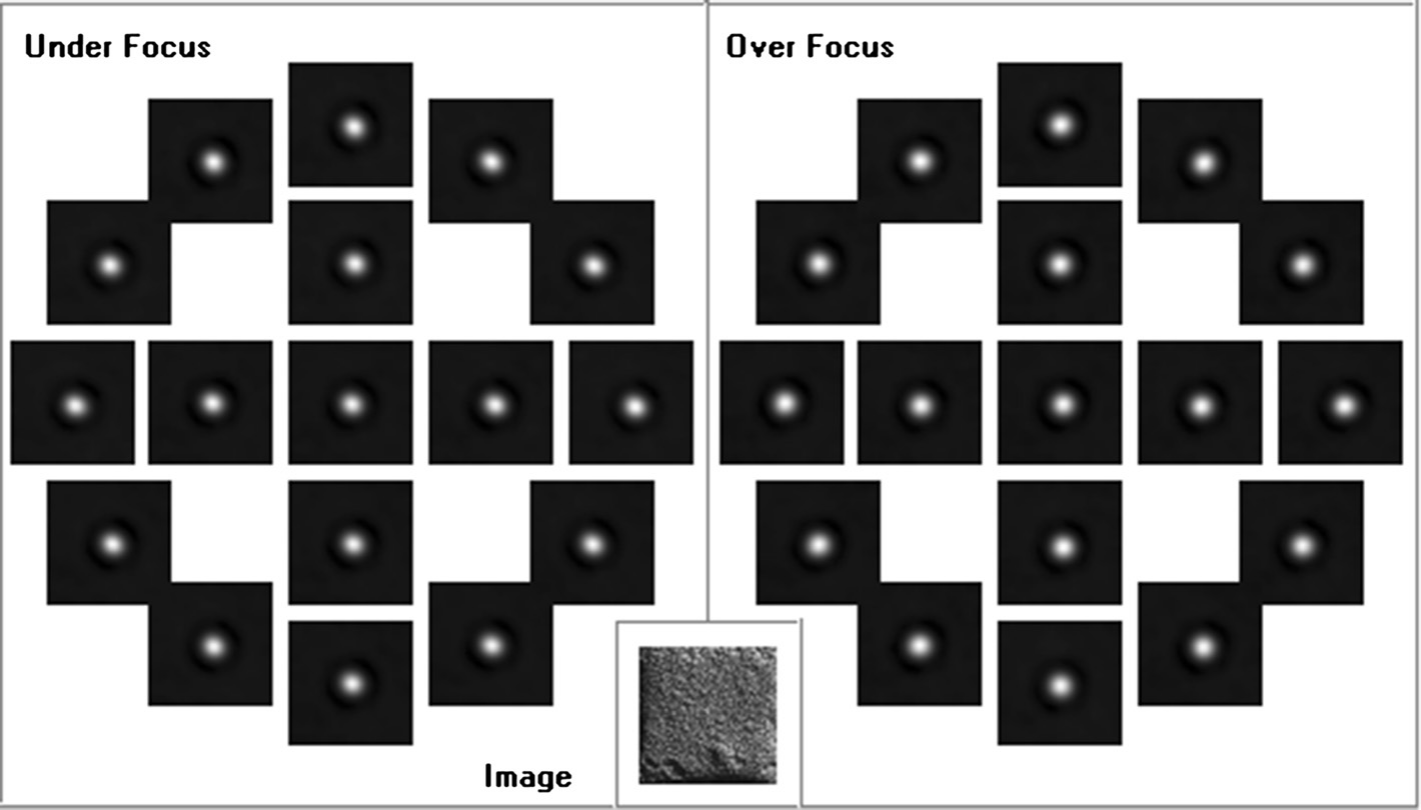
Zemlin tableau for measurement of aberration coefficients.

Figure 1 shows the Zemlin tableau, which consists of an initial Gaussian image and deconvoluted under- and over-focused probe shapes with a tilted incident beam of 25 mrad. The measurements were taken with varying values for the ‘outer tableau tilt’ *θ*_0_. The first tilt was performed in positive image x-direction (0°), and then, the azimuth was changed by -30° (clockwise) at each step. At an inner circle with a radius of 0.5 *θ*_0_, measurements were obtained at azimuths of 0°, -90°, 180°, and 90°. Analysis of the aberrations up to the five-order astigmatism *A*_4_ was preselected.

**Figure 2:**
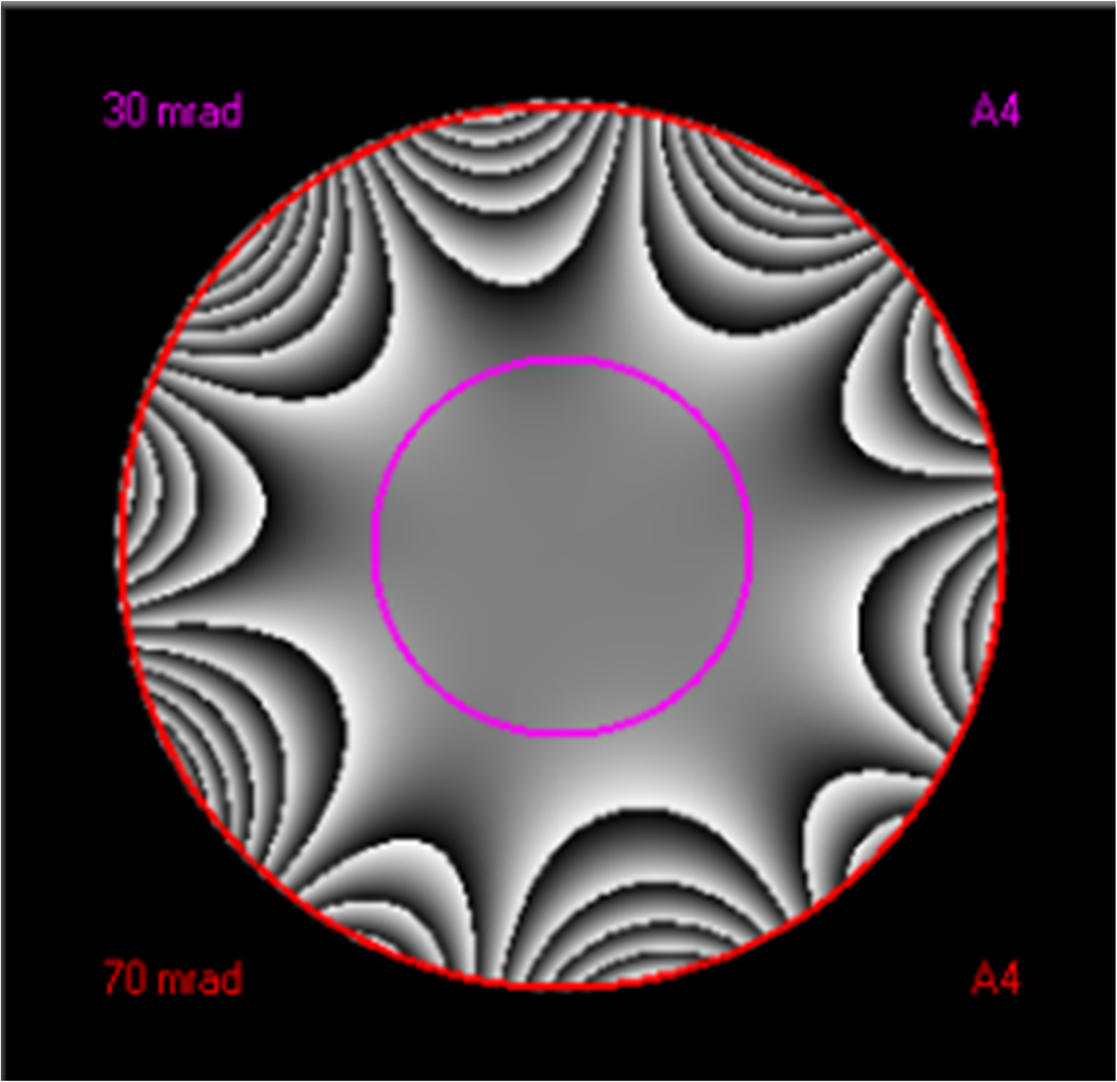
**Phase plate calculated from the aberration coefficients of the last measured probe tableau.** For a 25-mrad outer tilt angle of the optical axis.

The phase plate was then calculated from the aberration coefficients of the last measured probe tableau for different outer tilt angles of the optical axis. Figure 2 shows the phase plate used to visualize the corrected aberration coefficients. Here, the defocus *C*_1_ and two-fold astigmatism *A*_1_ were neglected. The black color indicates the upper limit towards - *π* + *k*2*π*, while white represents the lower limit towards + *π* + *k*2*π*. where π represents an integer. The phase plate was drawn to the tilt angle at which a limit of 12 π was reached (outer circle). The inner circle indicates the π/4 limit. The radii are displayed in the upper (30 mrad) and lower (70 mrad) left edges, respectively. Together with each radius, the five-order astigmatism aberration coefficient (*A*_4_) with the largest contribution at this radius is given on the right. The phase shift image (phase plate) was calculated from the measured aberrations for the DCOR. The approximate size of the corrected area is indicated by the 30-mrad (half angle) aperture.

For aberration correction, improvement in the transfer function was directly seen in the ronchigram, a diffraction pattern of an amorphous film obtained using a large convergence angle that gives a direct image of the aberration function *χ*(*θ*, *φ*). In the present aberration corrected system, there was a large uniform region at the center of the ronchigram. The range of minimum contrast at the center defined an area of constant electron phase that was appropriate for use in forming a small probe. By allowing higher convergence angles, the beam intensity also increased, which is important for chemical analysis.

### HRTEM characterization

**Figure 3:**
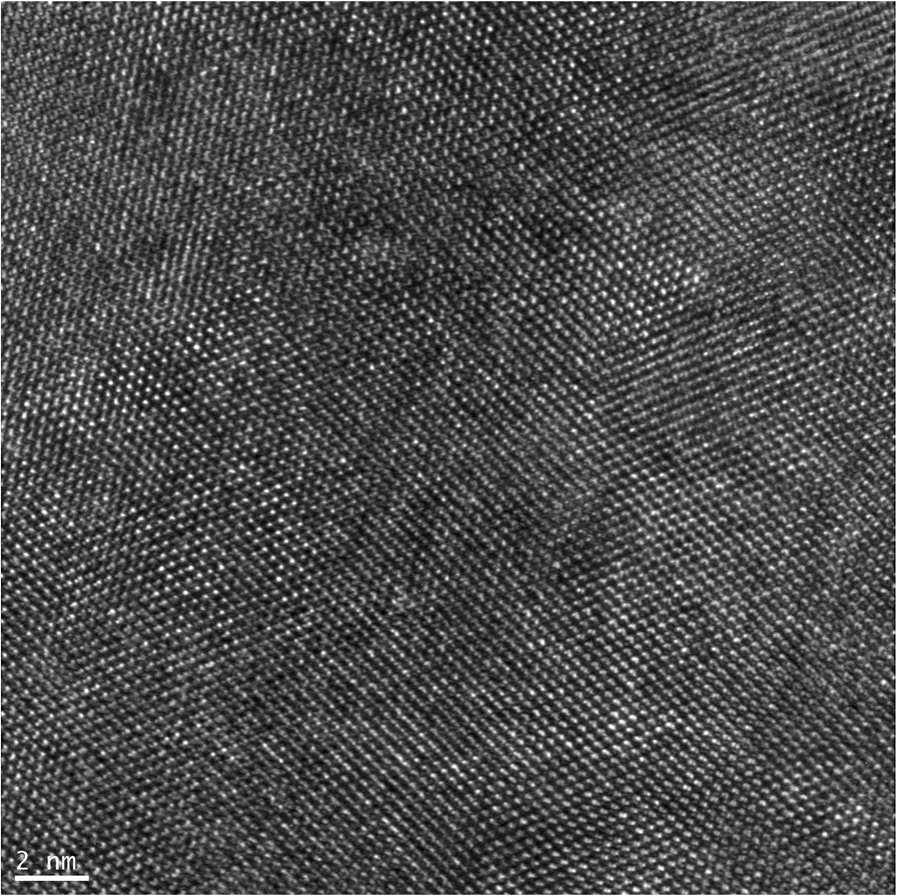
HRTEM image of the SiGe/Si interface.

The HRTEM image revealed that the interplanar spacing of the silicon (111) planes observed along the [110] zone axis was approximately 0.313 nm (Figure 3). The atomic structure of the SiGe/Si interface was not apparent, however, and thus, the SiGe/Si interface could not be identified using the phase contrast image formation. While the bright point indicated the intersection of the lattice fringes, the accurate position of the atomic column could not be determined.

### HRSTEM characterization

**Figure 4:**
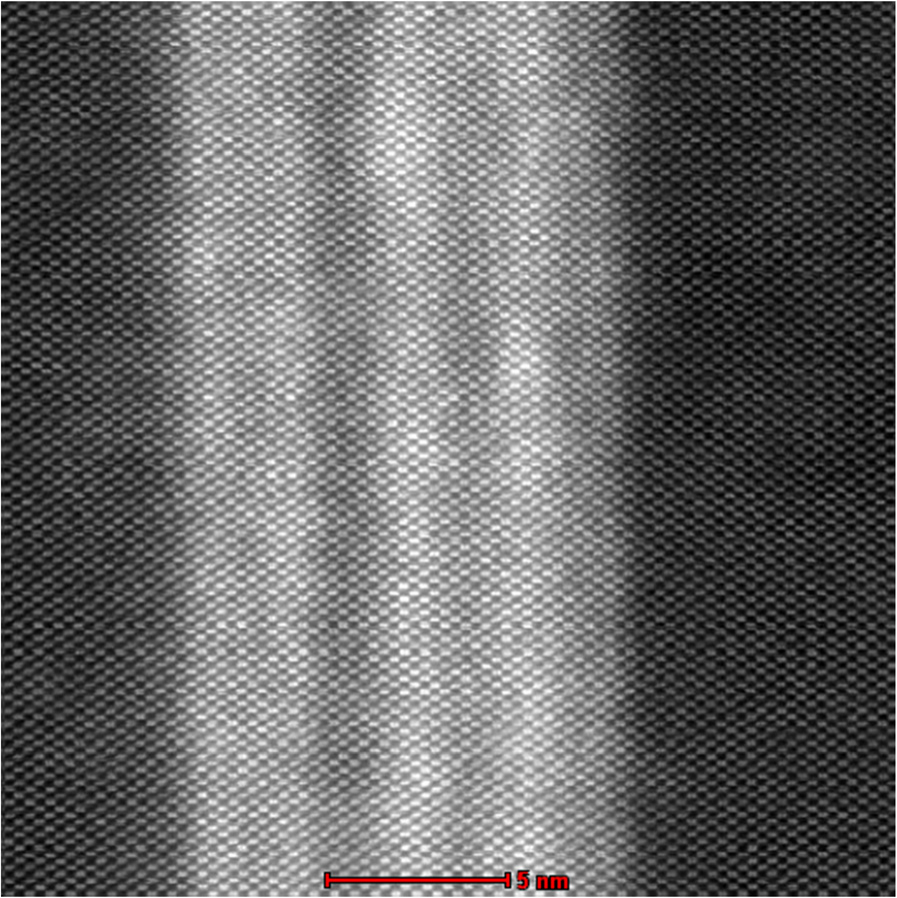
**HRSTEM HAADF image (*Z *** **contrast) of a 10-nm-thick SiGe epitaxial layer with the SiGe/Si interface.**

The HRSTEM analysis with a large coherent convergent beam provided incoherent atomic resolved images on a low-order zone axis through thermal diffuse scattering (TDS). In the high-resolution HAADF images, unlike for the HRTEM images, the projected atomic columns were identified by bright spots independent of the defocus of the probe-forming lens and the sample thickness, except for the unsuitable defocus values. In addition, the incoherent images displayed a contrast that was strongly dependent on the atomic number (*Z* contrast). Figure 4 presents the HAADF image of a 10-nm-thick SiGe epitaxial layer with an interface composed of a silicon crystal connected coherently to the superlattice of the SiGe layer. As can be seen in the figure, the contrast for the Si and SiGe atoms was clearer at the interface. Furthermore, the absence of the Fresnel interference effect and the high sensitivity to the tilting of the crystal made it easy to perform structural and compositional analyses at the interface and the surface region with atomic-scale resolution. Due to these advantages, the HAADF method is widely used for the analysis of boundary structures and for the distribution analysis of dopants in materials.

**Figure 5:**
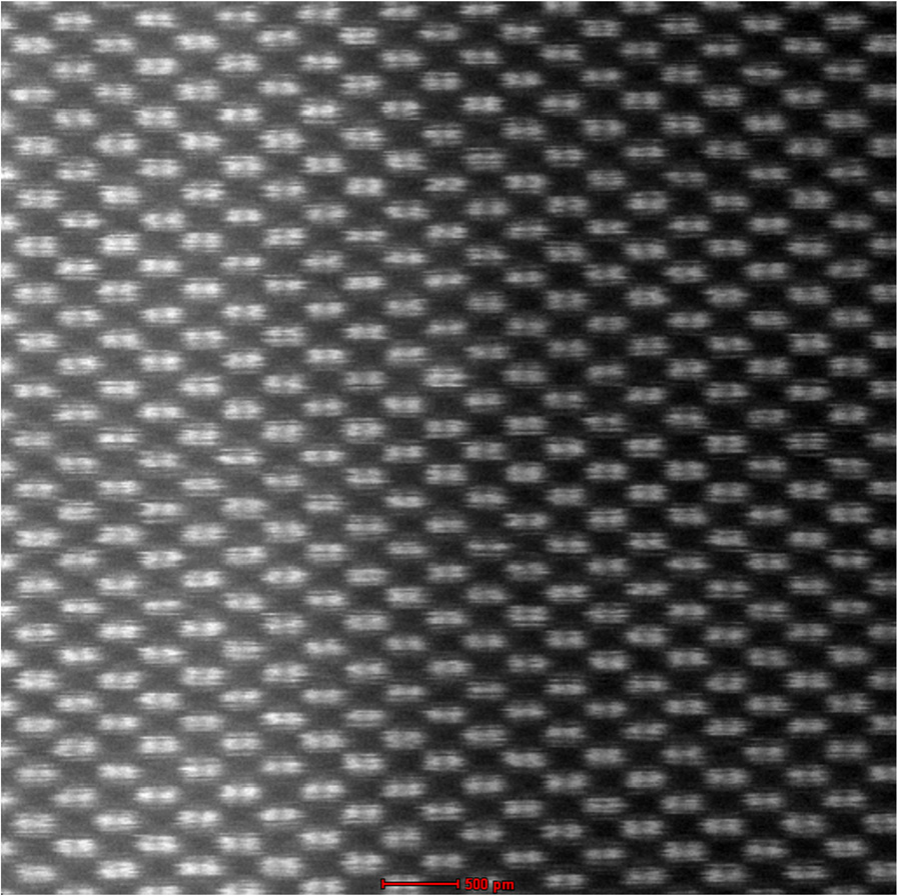
HRSTEM HAADF image of the dumbbell SiGe/Si interface.

**Figure 6:**
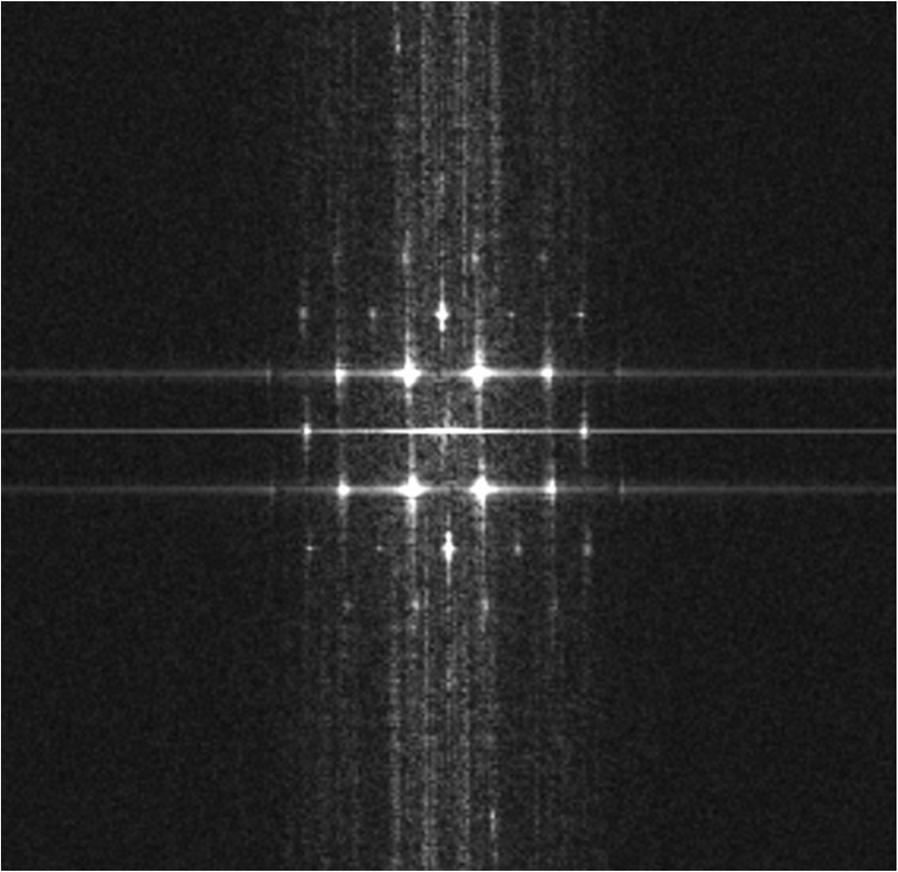
FFT result showing information transfer to 0.78 Å.

The resolution of such HRSTEM images can be determined using the criterion separation of the atom peaks in the image. Evaluation of the resolution using this criterion requires a specimen that can be tilted at an orientation in which the atoms are separated by a known distance. Figure 5 shows the HRSTEM dumbbell image of the SiGe/Si interface in the [110] orientation, and Figure 6 shows the fast Fourier transform (FFT) indicating a resolution of 0.78 Å (point resolution). The distance between the separated dumbbell Si and Ge atoms was approximately 1.36 Å. Models for silicon in the [112*n*] orientation show typical ‘dumbbell’ arrangements. Increasing the value of *n* from 0 to 3 moved the atoms closer together in the projection, and the projected atom-atom spacing in the silicon changed from 1.36 Å for the [110] orientation to 0.31 Å for the [116] orientation [16].

### EDS analysis

**Figure 7:**
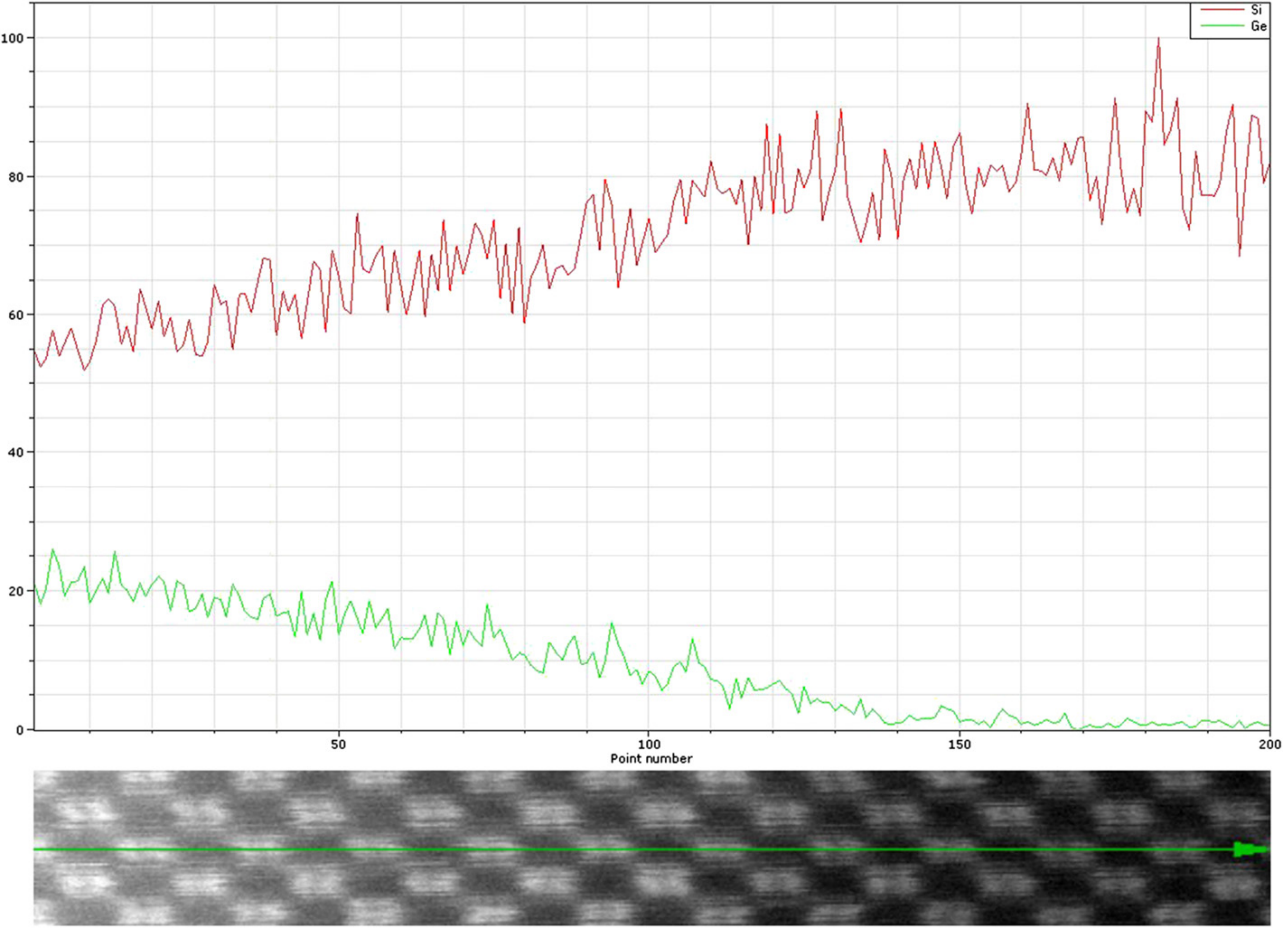
**Reference atomic resolution SiGe/Si interface HRSTEM HAADF image along the [110] direction.** With the corresponding EDS line scans for Si and Ge (red and green lines, respectively).

**Figure 8:**
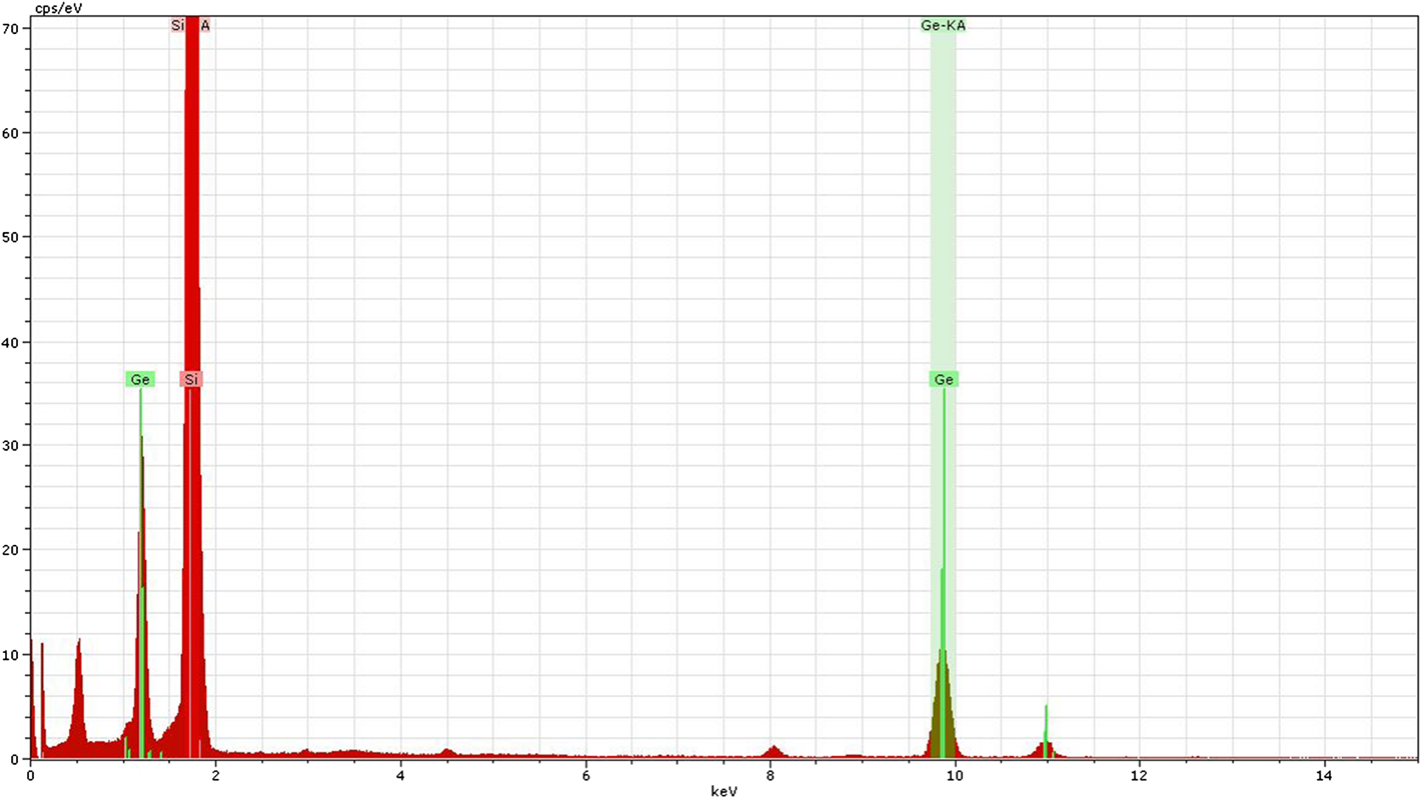
The integrated peaks for Si and Ge in the EDS spectrum.

Figure 7 presents the atomic resolution SiGe/Si interface HAADF image along the [110] plane with the simultaneously recorded EDS line scans (Si: red line, Ge: green line). The inelastic interaction of x-ray was effectively local, as was observed in the HRSTEM HAADF image, and the inner-shell ionization potential was as localized as possible for a given ionization edge. Consequently, it was possible to directly correlate the resulting incoherent HAADF image to the EDS line scan signals [15]. The interfacial layer contained a few atomic percent of Ge, the atoms of which had been diffused from crystalline SiGe nanolaminates. The EDS spectrum for Si and Ge is shown in Figure 8. The Ge line scan (green) was constructed from the sum of the Ge K- and L-shell ionization events. The corresponding Si line scan (red), however, was constructed using only the Si K shell. The detectors had count rates an order of magnitude greater than conventional detectors, and thus, the signal-to-noise ratio was improved.

## Conclusions

The atomic resolution imaging and spectroscopic analysis of an SiGe/Si interface grown via molecular beam epitaxy was investigated using aberration-corrected HRSTEM. The phase plates were calculated from the aberration coefficients of the measured probe tableau for various outer tilt angle of the optical axis, and the accuracy required for the compensation of the various residual aberration coefficients in order to achieve sub-angstrom resolution with the electron optics system was evaluated. The HRSTEM HAADF dumbbell image revealed that the distance between the Si and Ge atoms was approximately 1.36 Å, and the corresponding fast Fourier transform confirmed a point resolution on the sub-angstrom scale (0.78 Å). In addition, the experimental results demonstrated that complementary EDS line scan signals could be directly correlated to the atomic-resolution HAADF image.

## Competing interests

The authors declare that they have no competing interests.

## Authors’ contributions

CNH and SYK designed the experiment and measurements. CNH and WCC executed the experiments. CNH and FYL examined the written report. All authors read and approved the final manuscript.
